# Access and reimbursement of ambulatory cardiac monitoring across Europe

**DOI:** 10.1093/ehjdh/ztaf102

**Published:** 2025-08-30

**Authors:** Giuseppe Boriani, Johannes Brachmann, Thorsten Lewalter, David J Wright, Patrick Badertscher, Chris P Gale, José Luis Merino, Helmut Pürerfellner, Gregory Y H Lip

**Affiliations:** Cardiology Division, Department of Biomedical, Metabolic and Neural Sciences, University of Modena and Reggio Emilia, Policlinico di Modena, Via del Pozzo, 71, Modena 41124, Italy; Medical School Regiomed, Regiomed-Kliniken Coburg Germany and University of Split School of Medicine, Split, Croatia; Department of Cardiology and Intensive Unit Care, Hospital Munich South, Peter Osypka Heart Center, Munich, Germany; Department of Cardiology, University of Bonn, Bonn, Germany; Liverpool Centre for Cardiovascular Science, University of Liverpool and Liverpool Heart and Chest Hospital, Liverpool, UK; Department of Cardiology, University Hospital Basel, University of Basel, Basel, Switzerland; Cardiovascular Research Institute Basel, University Hospital Basel, Basel, Switzerland; Department of Cardiology, Leeds Teaching Hospitals NHS Trust, Leeds, UK; Leeds Institute of Cardiovascular and Metabolic Medicine, University of Leeds, Leeds, UK; La Paz University Hospital, Universidad Autónoma de Madrid, Idipaz, Madrid, Spain; Cardiology, Hospital Viamed Santa Elena, Madrid, Spain; Department of Electrophysiology, Ordensklinikum Linz Elisabethinen, Linz, Austria; Liverpool Centre for Cardiovascular Science at University of Liverpool, Liverpool John Moores University and Liverpool and Heart and Chest Hospital, Liverpool, UK; Danish Center for Health Services Research, Department of Clinical Medicine, Aalborg University, Aalborg, Denmark

**Keywords:** Reimbursement, Ambulatory Cardiac Monitoring, Innovation, Equitable Access

## Abstract

Ambulatory cardiac monitoring (ACM) allows long-term electrocardiogram (ECG) monitoring to detect arrhythmias with different modalities, ranging from short-term Holter monitoring (up to 48 h) to long-term continuous patch ECG monitors (up to 14 days), external event recorders (up to 30 days), and implantable loop recorders (ILRs). Access and reimbursement for ACM across Europe are not well understood. We performed a systematic review and analysis to understand ACM reimbursement across Europe, including a review of the reimbursement systems in each country and a detailed inspection of clinical coding and provider reimbursement. Level of reimbursement is dependent on many factors, including clinical setting (inpatient, outpatient, and day case), hospital length of stay, diagnosis, complications/severity, geographical location, hospital type, and device model and manufacturer. In most countries, reimbursement is performed for the monitoring procedure itself, without considering the time extension of monitoring and the specific type of device used for monitoring. The monetary value of reimbursement varies by country for both ACM and ILR [for Holter from €17.49 to €939.78 and for ILR from €416.14 (provider reimbursement only) to €18,718 (provider reimbursement bundled with ILR device)]. Holter and ILR are universally reimbursed, but newer ACM technologies with extended duration of monitoring, including long-term continuous monitoring and event recorders, are not. Across Europe, we found large variation in monetary values for reimbursement for ACM and ILR. We also found limited reimbursement and access to longer-duration ACM technologies. These findings suggest heterogeneous and problematic access to evidence-based tools for longer-duration monitoring.

## Graphical Abstract

**Graphical Abstract:**
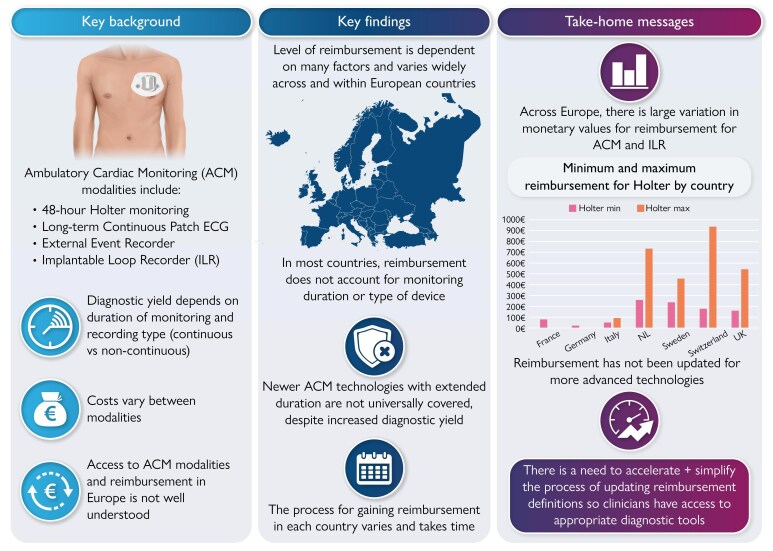

## Introduction

Ambulatory cardiac monitoring (ACM) is a key diagnostic tool, and traditional Holter monitoring has been the mainstay of ACM, providing data for up to 48 h. Event recorders, developed soon after traditional Holter monitoring, were designed to provide a longer wear time, but only recorded specific auto-detected events in a memory buffer that could be overwritten due to limitations of storage.

The classical technology of Holter monitoring was developed over a half-century ago and over the past few decades, there have been steady advances in the technology of ACM.^1–3^ The advances in ACM include longer storage for continuous recording, new form factors and biomaterials for uninterrupted recording and a simplified experience (patches), cellular transmission (mobile cardiac telemetry), and artificial intelligence for semi-automated arrhythmia extraction and classification.^3–6^ Costs vary considerably according to the complexity of the monitoring. As is known, the cost of an implantable loop recorder (ILR), including the implant procedure and monitoring for up to 5 years, is much higher than the cost of a Holter or patch ECG monitoring.^1^

Arrhythmia diagnostic yield depends on the frequency of symptoms and the duration of monitoring, which is reflected in practice. Traditional short-term Holter monitoring has been shown to have a lower diagnostic yield than long-term monitoring, with a diagnostic yield of 77–88% in women and 71–93% in men after 14 days of monitoring vs. yields of 11–48% and 26–59% after 48 h, depending on the type of arrhythmia.^7^ Other factors impacting on diagnostic yield are recording type (continuous/intermittent), how the data are reported (continuous/episodic), and activation of recording (automatic/patient-activated). Observational data have shown that newer technologies, particularly those with patch-based, long-term uninterrupted recording (usually 14 days) are associated with greater arrhythmia diagnostic yield,^8–12^ decreased odds of repeat monitoring, and lower incremental health care utilization.^13^

Access and reimbursement to all these different modalities across Europe have not been well characterized, despite guidelines supporting the use of specific modalities in common clinical scenarios. Therefore, we performed a review of reimbursement for ACM in France, Germany, Italy, the Netherlands, Sweden, Switzerland, and the UK to gain a deeper understanding of the reimbursement landscape from a public healthcare system perspective and to elucidate whether reimbursement reflects recent advances in ACM technologies.

## Methods

The adoption of clinically proven, regulatory-approved, innovative medical technologies relies upon three potential economic scenarios; that the public health system is sufficiently contemporary to reimburse the healthcare provider, that patients have access to private medical insurance or are willing to ‘self-pay’ or ‘pay-out-of-pocket.’

Our work focused on ACM (most data are on Holter, and to a lesser extent patch ECG monitors) and ILR. For each country, research was carried out by European Med Tech and IVD Reimbursement Consulting Ltd. (MTRC) to understand the provider reimbursement of ambulatory ECG monitoring and ILR, from a high-level review of the reimbursement system in each county to a more detailed inspection of clinical coding. The information was assessed to determine the granularity of the classifications available within clinical coding and each code’s corresponding reimbursement tariff.

The data for reimbursement analysis in selected European countries were obtained after reviewing the latest versions of reimbursement principles published by the responsible governmental authorities and supporting agencies: Liste des Produits et Prestations Remboursables (LPPR) from the Haute Autorité de Santé (HAS) in France, Federal Ministry of Health (Bundesministerium für Gesundheit) and National Association of Statutory Health Insurance Physicians (KBV) in Germany, Servizio Sanitario Regionale Ministero della Salute in Italy, Dutch Healthcare Authority (NZa) in the Netherlands, National Board of Health and Welfare (Socialstyrelsen) in Sweden, Federal Office of Public Health in Switzerland and National Health System Payment Scheme in the UK.

The scope of the analysis included reviewing all relevant procedure codes for the ILR, which included implantation, replacement, and removal, both as an inpatient and as a day case procedure. The same approach was used for ambulatory ECG monitoring, but was confined to the outpatient setting.^14–21^

In addition, the latest versions of diagnosis coding nomenclatures for different arrhythmias, including atrial fibrillation, syncope, other supraventricular cardiac arrhythmias and ventricular arrhythmias were considered during the analysis.^21–30^

Reimbursement tariffs for ILR were considered for regular hospital admission and day case procedures by combining procedure and diagnosis codes in each health system’s grouping software for Germany, Netherlands, Sweden, Switzerland, and the UK^31–38^ Further to the published unit price for each coded activity in the UK, these tariffs are also subject to the ‘market forces factor’ (MFF), which is a multiplier adjustment paid in addition to those national tariffs based on the cost of living within a given geography.^39^ For Italy, benchmark tariffs were available from the Ministry of Health^40,^ and local tariffs were publicly available from select regions’ local health authorities.^41–43^ For France, reimbursement tariffs are published on the French National Authority for Health^44^ and the LPPR information systems.^45^ Provider reimbursement tariffs for ILR only include professional fees and not the device cost, with the exception of the UK which include ILR device costs in the reimbursement tariff.

In France, the French National Authority for Health and the National Health Insurance Fund publish reimbursement tariffs for ambulatory ECG monitoring.^46–48^ In Italy, Emilia Romagna, Veneto, and Lombardy regional health authorities publish reimbursement tariffs for ambulatory ECG monitoring.^49–51^ In Sweden, reimbursement tariffs for ambulatory ECG monitoring were sourced from the Swedish Association of Local Authorities and Regions.^52^ In addition, the 2024 Diagnosis Related Group (DRG) tariffs were obtained by multiplying the national prospective DRG cost weight by the national base tariff for outpatient medical services.^53^ In the UK, the reimbursement tariffs for ambulatory ECG monitoring were obtained from the national payment scheme, with the unit price for the activity being subject to the MFF.^31,39^

In Germany, the Netherlands, and Switzerland, different sources were used for the analysis of reimbursement for ambulatory ECG monitoring. In Germany, reimbursement tariffs for ambulatory ECG monitoring were obtained in the 2024 version of the national catalogue for outpatient medical services. The 2024 DRG tariff was calculated as a sum of the tariff for the case and the tariff for nursing care, which differs depending on the length of stay.^54^ In the Netherlands, the weighted average price for ambulatory ECG monitoring, performed at the request of primary care specialists, was calculated using the 2024 pricelist of three Dutch University Medical Centers.^55–57^ In Switzerland, reimbursement tariffs for ambulatory ECG monitoring and ILR were calculated using the point value of individual procedures in the national catalogue for outpatient medical services.^58,59^

Where required, the currency was exchanged to € using the following exchange rates: £ to €1.19; CHF to €1.06 and SEK €0.088, which were the rates on 29 September 2024.

## Results

The results are divided into four sections: overview of available ECG monitoring methods, funding of public healthcare, the process of gaining reimbursement and reimbursement itself.

### Overview of available ECG monitoring methods

*Table 1* details the available ECG monitoring methods and their place in diagnostics and patient management, together with details of the different approaches and devices.^1,4^

**Table 1:** Available ECG monitoring methods

Method	Holter	Patch ECG monitor	External loop and event recorder	ILR
Monitoring time	24–48 h	Up to 14 days	Up to 30 days	Up to 5 years
Recording type	Continuous interrupted	Continuous uninterrupted	Loop	Loop
Data reported	Continuous	Continuous	Episodic	Episodic
Monitor activation	Always active	Always active	Must be activated by the patient	Automatically activated when arrhythmia detected/patient activated
Patient sub-group	Traditional diagnostic tool for patients with frequent (daily) arrhythmias	Appropriate for all patients with daily, weekly, or fortnightly arrhythmias including patients currently prescribed Holters	Appropriate for patients with infrequent and less critical symptomatic arrhythmias	Reasonable/recommended option for AF detection in patients with recent cryptogenic recurrent syncope of uncertain origin
Comments	Utility limited by short monitoring period and low diagnostic yield Electrodes need frequent changing, and this can lead to less continuous wear time	Found to be more sensitive than 24-hour Holter monitor, with better patient adherence	Preferred for patients with infrequent symptomatic events Electrodes need frequent changing, and this can lead to less continuous wear time	Most expensive and invasive solution only appropriate for the highest-risk patients Recordings are not continuous Requires patient adherence with gateway or smartphone to communicate symptom occurrence and alerts

There are differences in monitoring time, recording type (continuous, non-continuous), how the data are reported (continuous, episodic), and how the monitor is activated (always on, activated by the patient, automatically activated by the arrythmia).

### Funding of public healthcare

Public healthcare in the seven countries varies both in terms of how it is funded (public, private, or mixed) and how hospitals are reimbursed for treatment (*Table 2*).

**Table 2:** Reimbursement in secondary care

Country	Healthcare system	Hospital reimbursement	Funding of ACM
France	Publicly funded	In the inpatient setting, DRG system with a DRG to cover hospital admission and add-on reimbursement for some procedures and devices French DRGs have four severity levels, depending on the presence of complications and length of stay In the outpatient setting, reimbursement is via a fee for service, in order to be reimbursed service must be included in the Common Classification of Physician Services	DRG (inpatient) or fee for service (outpatient)
Germany	Mixed, 90% funded by public health insurance contributions shared by employees and employers Some elements of care (∼10%) require a top up charge if they are not included in the EBM	In the inpatient setting, DRG system with a DRG to cover hospital admission and add-on reimbursement for some procedures and devices In the day case and outpatient setting, reimbursement is via a fee for service, in order to be reimbursed service must be included in the EBM	DRG (inpatient) or fee for service (outpatient/day case)
Italy	Publicly funded, administered regionally	Reimbursement depends on ownership status of hospitals Aziende Ospedaliere hospitals are quasi-independent hospital companies which use DRGs to invoice local health authorities (ASLs) ASL-run hospitals are allocated a global budget from the ASL to pay for services provided Regions can introduce add-on reimbursement, for the device or a specific procedure, and DRG split, in order to adequately finance the procedure Some procedures are reimbursed on a fee for service basis	Fee for service model based on ICD-9-CM procedure codes Each region (*n* = 21) has a different nomenclature and tariff for their outpatient services
Netherlands	Mixed, funded by payroll taxes (50%), government (5%), and compulsory private insurance (45%)	DRG, either national (A) or free pricing (B) determined by negotiations between insurance companies and hospitals (about 80% of DRG) Dutch DRGs typically cover periods beyond hospital admission (e.g. typically include admission and several outpatient visits/manipulations covering a 4-month period) Some specialist services known as supplementary services (overige zorgproducten) are reimbursed via a fee-for-service model (either separately or in addition to a DRG)	DRG B or fee-for-service model
Sweden	Publicly funded, 21 regional payers	Reimbursement for hospital and outpatient specialist procedures is made through a combination of a global budget and DRG-based payment. Sweden uses the NordDRG system, which is shared with other Nordic countries Some regions are funded via the global budget and others via DRG	DRG, specific DRG for inpatient and for outpatient services Reimbursement is performed for a procedure. Brands of devices are not reflected in the system
Switzerland	Mandatory for citizens to purchase private social health insurance, Federal and Cantonal governments provide subsidy to low income citizens	DRG with add-on reimbursement for expensive procedures, pharmaceuticals and devices Variable charge, multiplied by Canton specific base rate to obtain Tarriff	DRG (inpatient) or fee-for-service model (TARMED) (outpatient)
UK	Publicly funded Private healthcare is optional	Delivered as part of an aligned payment and incentive Specific activity codes (Office of Population Censuses and Surveys Classification of Interventions and Procedures, OPCS) map through to DRG DRG tariff prices are subject to MFF, a fixed percentage uplift applied to the unit price depending on the trust, varies from 1% to 21% Reference costs include staff, outpatient clinic staff, estates, clinical supplies and services, hardware, software, and overheads In cases where the annual contract value between the commissioner and the provider, is <£0.5 m—a ‘LVA block payments’ mechanism applies	DRG

Regardless of the overall funding, public healthcare for secondary care across Europe is generally reimbursed using either a DRG model or a fee-for-service model. There is an added layer of complexity in that ACM is funded in different ways across the seven countries, often via DRG if provided as an inpatient and via fee-for-service if provided as an outpatient or day case, although there is some variation.

### The process of gaining reimbursement

Reimbursement is a two-step process. First, health technology assessment (HTA) is carried out to ensure that any new technology is both clinically and cost-effective.^60,61^ Once HTA approval has been gained, then reimbursement systems must be updated to reflect the new technology. Efficiency in updating reimbursement systems varies widely across Europe; however, most are cumbersome and slow to update reimbursement to reflect technological advances.

HTA bodies vary in their remit across Europe, some countries have a specific HTA body for devices (United Kingdom, Sweden, Wales), whilst other countries include HTA within their national/regional HTA bodies.^62^ However, new devices do not automatically enter the HTA process; scoping and the decision to include a new device in an HTA program can be lengthy. Once accepted into the HTA program, the HTA process itself can take up to 1 year from submission of the manufacturer’s HTA dossier, see *Table 3*.

**Table 3:** HTA process for medical devices

	HTA for devices	National/Regional	Timelines from submission to approval (estimated values)
France	Yes	National	180 days
Germany	Yes	National	3–6 months
Italy	Yes	Regional	Variable
Netherlands	Yes	National	18 weeks
Sweden	Yes^a^	Regional/National	Variable
Switzerland	Yes	Regional	6–12 months
UK: England	Yes^a^	National	32 weeks
UK: Scotland	Yes^a^	National	6–9 months
UK: Wales	Yes^a^	National	Variable

Modified from Ref.^62^

^a^Specific HTA body/bodies for devices/procedures.

A review of published and ongoing HTAs of implantable and wearable devices for ACM in each country is included in Supplementary material online, Results. HTAs for cardiac monitoring were rare in most countries, with the exception of Sweden and the UK. Notably, HTAs for implantable devices were common in France, but there were no published or ongoing HTAs for ACM.

A brief outline for gaining reimbursement post-HTA in each country is provided in the following.

In France, the manufacturer can apply for inclusion in the LPPR, which provides access to add-on reimbursement. At least one good quality randomized controlled trial is required, and a price must be negotiated nationally. This process can take up to 2 years. However, in order to gain reimbursement, a Classification Commune des Actes Médicaux code is required, and it can take up to 5 years to obtain a code.

In Germany, inpatient reimbursement is via DRG, which is updated each year. The updates are based solely on an analysis of historical cost data from 15% of German hospitals. There is additional flexibility in that hospitals can make suggestions for DRG change to the Institute for the hospital remuneration system. For outpatient reimbursement, the procedure must be in the Einheitlicher Bewertungsmaßstab (EBM) catalogue. In order to enter the EBM catalogue, new diagnostics and treatments must be assessed by the Evaluation Committee (Bewertungsausschuss), a process which typically lasts around 2 years.

In Italy, guaranteed health benefits to the population are defined in the essential levels of care, however, it provides only a vague description of guaranteed hospital services and reimbursement is on a regional basis. Updating reimbursement is challenging and DRGs were last updated nationally in 2012. Local add-on reimbursement can be introduced, for a device or specific procedure, although the pathways to engender change are inconsistent and include local lobbying with the provision of clinical and health economic supportive data.

In the Netherlands, Basic Health Insurance is determined/guaranteed by the Health Insurance Law (Zorgverzekeringswet [Zvw]), which describes care in very general terms, meaning that there is a lack of clarity regarding what is covered. The NZa is responsible for the development of procedural classifications and an update of the DRG system. Routine updates of the system are based on both analysis of cost and suggestions from providers and insurers.

In Sweden, there are three key elements for market access for medical technologies: reimbursement (payment mechanism via the DRG-adjusted budget system), funding (recommendations in the national Orderly Introduction of Medical Technologies framework), and HTA (obtaining recommendations from the national [Swedish Council on Technology Assessment in Healthcare] and regional HTA bodies). DRGs are determined by the combination of a procedure code (Klassifikation av vårdåtgärder [KVÅ]), which are updated annually, and a diagnosis code (ICD-10). Applications for a new procedure code and for DRGs are made by physicians and take 1 year to complete. In general, HTA recommendations are not mandatory for hospitals to follow; however, they can be impactful as Swedish physicians are typically very evidence-based.

In Switzerland, all inpatient procedures are considered eligible for coverage within the Swiss mandatory health insurance. However, insurance companies and hospitals can question medical procedures, which then must undergo assessment by the Federal Office of Public Health for approval, which has a reasonably rapid turnaround. However, the current TARMED tariff, which reimburses outpatient ACM, has not been updated since 2018 and will be updated in 2025 when it is replaced by a new TARDOC tariff.

In the UK, the National Health Service is legally bound to fund and resource devices approved by the National Institute for Health and Care Excellence, and the bodies in Scotland (Scottish Health Technologies Group) and Wales (Health Technology Wales) provide advice to inform decision-making. However, OPCS codes for operations and procedures are only updated periodically, every two to three years, with the latest OPCS codes published in 2023.

### Reimbursement of ACM

The cumbersome and lengthy procedures for defining reimbursement that occur in many systems across Europe mean that not all types of ACM are reimbursed within the seven countries, as shown in *Table 4*. Holter and ILR are universally reimbursed, however, newer technologies such as ECG patches and external recorders are not, meaning that reimbursement does not reflect recent advancements in ACM technologies. In most countries, reimbursement is performed for a procedure and the different characteristics and performance of available devices are not reflected in the system.

**Table 4:** Availability of reimbursement of ACM

	Holter	Patch	External loop and event recorder	ILR
France	Yes	No	Unclear	Yes
Germany	Yes	No	Unclear	Yes
Italy	Yes	No	Unclear	Yes
Netherlands	Yes	Unclear	Unclear	Yes
Sweden	Yes	No	Unclear	Yes
Switzerland	Yes	No specific code	Yes	Yes
UK	Yes	No specific code	Yes	Yes

Reimbursement varies in complexity across the countries and according to the method of reimbursement. A detailed table providing information on reimbursement methods, coding, and fees is provided in Supplementary material online, Table.

For example, in the UK, one DRG covers seven OPCS codes for ambulatory ECG monitoring, whereas in the Netherlands, there are two specific DRG codes for Holter monitoring used in combination with different allocations depending on medical specialty and diagnosis, and in Germany, ambulatory ECG monitoring is reimbursed via fee-for-service with codes for each element of the service.

The level of reimbursement is dependent on many factors and varies by country and even within country. The factors conditioning the level of reimbursement include length of stay, setting (inpatient, outpatient, day case), diagnosis, complications/severity, geographical location, hospital type, and brand of device. Details are shown in *Table 5*.

**Table 5:** Factors impacting on reimbursement of Holter and ILR across Europe

	France		Germany		Italy		Netherlands		Sweden		Switzerland		UK	
	Holter	ILR	Holter	ILR	Holter	ILR	Holter	Holter	Holter	ILR	Holter	ILR	Holter	ILR
Setting (inpatient/out patient)						✓	✓		✓	✓			✓	✓
Diagnosis (underlying heart disease)	✓	✓	✓	✓			✓		✓	✓^a^				
Complications/ severity of diagnosis		✓		✓	✓	✓			✓^b^			✓		✓
Length of stay		✓		✓	✓	✓						✓		
Type of monitoring					✓						✓			
Brand of device				✓										
Geographical location (regional tariffs/regulations)	✓	✓				✓	✓				✓	✓	✓	✓
Hospital type (private/public)				✓										

^a^Outpatient only.

^b^Inpatient only.

The Swiss approach to Holter monitoring is interesting since it reimburses the procedure itself—with specific codes for ECG registration (including connection to the device and its removal) and codes for assessment of results, but also considers the duration of monitoring (up to 48 h, 48 h to 6 days, and >6 days) and the cost of the consumables. Consumables and materials, i.e. the monitor, may be billed separately if the purchase price (including VAT) exceeds 3 CHF per item—which provides an opportunity for new devices and modalities to be used.

**Figure 1:**
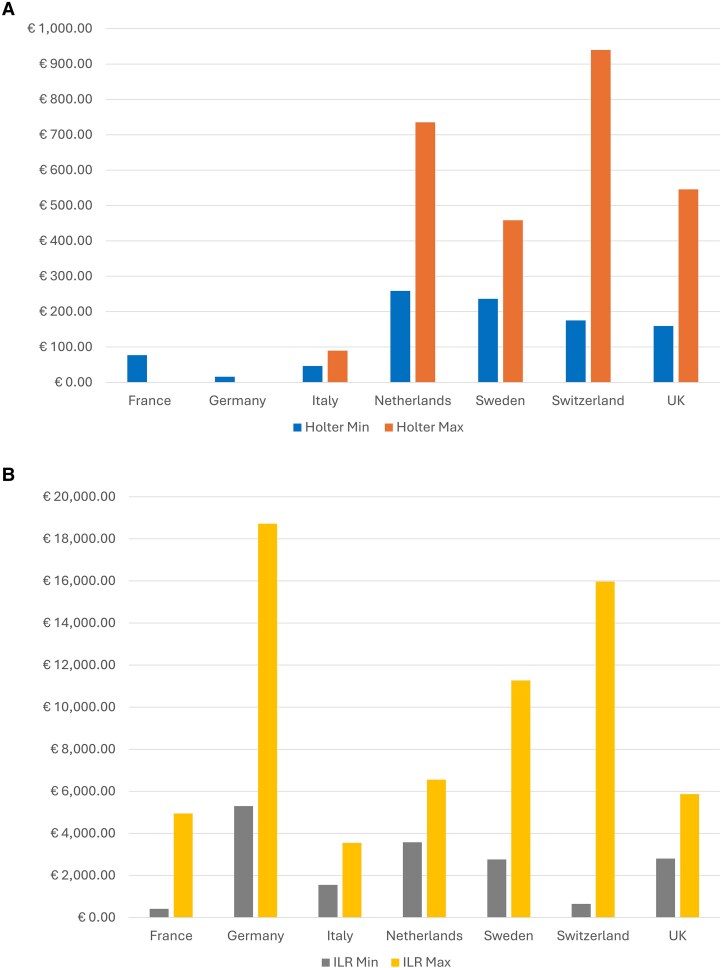
Minimum and maximum reimbursement for Holter (A) and ILR (B) across multiple settings.

Heterogeneity in the monetary value of reimbursement for both ACM and ILR is clear when the monetary value of the reimbursement is considered. *Figure 1* shows the minimum and maximum reimbursement fees for the countries that we analysed. The differing characteristics and financial profile of the countries should be taken into consideration, even so the extent of variability is considerable. The monetary value of reimbursement varies by country for both ACM and ILR (for Holter from €17.49 in Germany to €939.78 in Switzerland and for ILR from €416.14 in France to €18 718 in Germany (details in Supplementary material online, Table). Some variability also exists within countries, as in the case of Italy (Supplementary material online, Table).

It was challenging to estimate these numbers from the reimbursement information, and it should be noted that there are several caveats. In France, there are additional fees that have not been included, therefore, the fee is likely to be higher. In Germany, day case implantation of ILR is not within the EBM, but removal is coded, with monitoring during the operation, anesthetic, post-operative monitoring, and care reimbursed separately. The minimum and maximum fees for day case removal of ILR are €113.84 and €249.29, respectively. Costs vary across Italy because reimbursement is carried out regionally, this data is the national fee so might be higher or lower depending on location. In the Netherlands, costs are the average from three University Medical Centers, meaning costs may be higher or lower in other hospitals within the Netherlands. In Switzerland, prices are exclusive of consumables in the TARMED catalogue, meaning that the cost of reimbursement in day case and outpatient settings is likely to be even higher, however, according to the healthcare benefit ordinance (KLV), the ILR implantation and removal procedures should be performed in day case and outpatient settings (with fee-for-service reimbursement via TARMED which equate to €1002.23). Finally, with the exception of the UK, the cost of the ILR device is not included in the provider reimbursement for outpatient ILR implantation, varies by country, and there is limited publicly available data (Supplementary material online, Table). Notwithstanding these limitations, together with any shortcomings in data collection and analysis and exchange rate variability it is absolutely clear that reimbursement is hugely variable, despite common clinical guidelines across Europe and comparable diagnostic pathways.

## Discussion

Our analysis highlighted large variations in monetary values for provider reimbursement of ACM and ILR across Europe. The disparities in reimbursement tariffs for specific modalities of ACM and traditional approaches and definitions for reimbursement have several implications for clinical practice. First, most reimbursement practices across the seven European countries only include traditional Holter, external patient-activated recorders, and ILR, and do not specifically include long-term continuous monitoring ACM devices, such as patch-based technologies. Therefore, clinicians are not able to apply, despite the available studies and clinical evidence, these non-invasive new technologies, which have proven diagnostic capabilities for capturing arrhythmias over a 14-day period in appropriate patients. It is proven that diagnostic yield improves with duration of monitoring, particularly in patients with infrequent arrhythmias.^7^

Secondly, a traditional Holter is often considered the sole diagnostic method to be applied in clinical practice, meaning that in many cases traditional Holter is used regardless of how the patient presents. Inopportune timing of clinic visits for conventional Holter monitoring may mean that arrhythmias are missed, resulting in adverse outcomes for patients, and it is often repeated in the hope that the patients experience their arrhythmia during their wear time, which is costly for both the health care system and the patient.^3^ In many settings, the reimbursement system provides a choice mainly focused on traditional Holter or ILR, with clear differences in terms of cost and acceptance. This commonly leads to repetition of traditional Holter with sequential costs for both the health care system and the patient.^3^ Although Holter monitoring has a relatively low cost in terms of set-up costs, it can be expensive in terms of cost per diagnosis if multiple Holter recordings are required. Selection of the most appropriate ACM is required to optimize arrhythmia diagnostic yield. Studies comparing traditional 24–48 h Holter with patch ECG have shown a significant increase in the number of arrhythmia events detected^8,9,12^ in patients referred for evaluation/suspicion of cardiac arrhythmia and in those with unexplained syncope.^63^ A large Medicare claims database retrospective cohort study in patients without a diagnosis of arrhythmia receiving ACM for the first time,^13^ revealed diagnostic yields of 33.8% for patch monitors with 14-day recordings, 22.7% for Holter, 24.6% for external event recorders, and 27.1% for mobile cardiac telemetry. In detail, 14-day ambulatory patch monitor had the highest diagnostic yield of all patch monitors, and when it was taken as the reference category, Holter, external event recorders, and mobile cardiac telemetry had significantly lower adjusted odds of new diagnosis (0.5–0.83) and higher adjusted odds of retesting (1.35–4.27), regardless of the specific arrhythmia diagnosed.^13^

Data indicate that patients express preferences for using a patch ECG; specifically, in one study 81% preferred patch ECG over traditional Holter monitoring, with almost all patients (94%) finding the ECG patch comfortable to wear, which has implications for patient adherence with monitoring. Furthermore, the current tendency to move from relatively long hospitalization times to 1-day surgery or even to an ambulatory regimen for many procedures in the field of arrhythmia management and electrophysiology^64^ may have an impact on the setting for prescription of ACM, with an increase in outpatient use of ACM.

Thirdly, disparity in reimbursement policies for ACM has hindered the widespread adoption of longer-term cardiac monitoring, such as non-invasive ECG patches, which should be considered as a new standard for patients whose symptoms are infrequent, avoiding the risk of missed diagnosis with clinical and financial consequences.^65^ Current reimbursement policies anchored to Holter monitoring devices do not reflect the increase in cost associated with advancements that enable improved outcomes. Holter monitors are reused and require that patients travel to the clinic to initially receive and then return the device after the wear period. Holter monitors have shorter monitoring durations, which results in less data to store, transmit, and interpret. Future pricing should consider factors, such as at-home application (including shipping to and from), use of disposable technology or cost of reprocessing, increased data handling costs with large volumes of ECG data, use of artificial intelligence and proprietary algorithms, ongoing operational and support costs, and review by certified technicians. The process of changing reimbursement, beginning with performing an HTA, can be lengthy. Many regions do not have a body equipped to review dossiers specific to medical devices. Few countries in the EU have systematic and centralized systems for review of HTA. Many countries limit review of HTAs to certain technologies or distribute responsibilities across multiple bodies or regions, which can lead to variation in access.

These findings are consistent with physician perceptions, recorded in a recent survey. The European Heart Rhythm Association (EHRA) issued a practical guide to the use of digital technologies to detect and manage arrhythmias, including ECG patches, wearable and handheld devices.^4^ A survey of 217 healthcare professional respondents from across Europe revealed that, despite a high level of trust in, and use of, digital technologies, there was a lack of reimbursement, limiting their use. Specifically, physicians were limited in their use of digital technologies, due to a lack of reimbursement for their consultation time for interpretation of results.^66,67^

## Conclusions

Across Europe, the wide variability in monetary values for reimbursement and the lack of updates to more advanced technologies for ACM after their validation in clinical practice suggest heterogeneous and problematic access to new and evidence-based diagnostic tools for longer-duration monitoring. There is a need for accelerating and simplifying the process of appropriate reimbursement definition, in order to enable clinicians to access all the appropriate diagnostic tools.

## Supplementary Material

ztaf102_Supplementary_Data

## Data Availability

Data are available upon reasonable request to the corresponding author.

## Lead author biography

Professor Giuseppe Boriani, MD, PhD, FEHRA, FESC, FHFA

Graduation in Medicine, Specialization in Cardiology, University of Bologna, Italy.

Ph.D. in Cardiovascular Physiopathology, University of Milan

Full Professor of Cardiology at the University of Modena and Reggio Emilia and Chief of Cardiology

Director of the Post-Graduate School of Cardiovascular Diseases, University of Modena and Reggio Emilia

Member of the Board of EHRA and Chair of the Health Economics Committee (2009-2013)

Editor-in-Chief of EP-Europace (since 1 July 2024)

Scientific activity has been focused on clinical cardiology, implantable defibrillators, atrial fibrillation, heart failure, telemedicine, digital health, and health economics
