# Supplementary material for: Access and reimbursement of ambulatory cardiac monitoring across Europe
Source: Eur Heart J Digit Health. 2025 Aug 30;6(6):1282–92. doi: 10.1093/ehjdh/ztaf102 (PMC12629646; doi:10.1093/ehjdh/ztaf102)
Supplement: ztaf102_Supplementary_Data [file ztaf102_supplementary_data.docx]

# **Supplementary Material**

| Supplementary Methods: Methodology for review of health technology assessments (HTAs) of ambulatory cardiac monitoring (ACM) in Europe. | Page 2 |
| --- | --- |
| Supplementary Results: Results for review of health technology assessments (HTAs) of ambulatory cardiac monitoring (ACM) in Europe | Page 4 |
| Supplementary Table: Reimbursement of ambulatory ECG monitoring and ILR | Page 19 |

**Supplementary Methods: Methodology for review of health technology assessments (HTAs) of ambulatory cardiac monitoring (ACM) in Europe**

The report includes the list of published and ongoing health technology assessments (HTAs) of ambulatory cardiac (heart rhythm) monitoring devices in France, Germany, Italy, the Netherlands, Sweden, Switzerland, and the United Kingdom (England, Scotland, and Wales) from January 1, 2015, to May 12, 2025.

The search was focused on HTAs of implantable and wearable devices for ambulatory heart rhythm (ECG) monitoring. The HTAs of devices for monitoring parameters other than heart rhythm, including blood pressure and pulmonary artery pressure monitoring, and cardiac implantable electronic devices with therapeutic function (cardiac pacemakers, ICDs, etc.) were excluded.

The search results in individual countries are presented in a table format.

In France, the assessments published by the National Authority for Health (HAS) were screened. HAS is a consultative body providing independent scientific advice to the French public authorities. It commissions the clinical evaluation of invasive medical devices and implants, procedure codes, and innovation funding decisions to the National Committee for the Evaluation of Medical Devices and Health Technologies (Credits) to support the decisions on add-on reimbursement via the List of Reimbursable Products and Services (LPPR). CNEDiMTS determines Actual Benefit (SA) of a device, which may be rated as sufficient or insufficient, the level of Added Clinical Value (ASA), and specific indications for reimbursement.

In Germany, the publications of the Institute for Quality and Efficiency in Healthcare (IQWiG) were screened. IQWiG is the main HTA body, functioning as an independent scientific institute evaluating the benefits and harms of medical interventions to support evidence-based decision-making in the statutory healthcare system. The evaluations are commissioned primarily by the Federal Joint Committee (G-BA), the highest decision-making body in healthcare in Germany, and by the Federal Ministry of Health (BMG).

In Italy, the National Agency for Regional Health Services (AGENAS) and regional HTA agencies (Lombardy HTA Program for medical devices and Tuscany Regional Working Group on Medical Devices) were screened. AGENAS conducts a comparative analysis of the cost and effectiveness of public health services, including evaluation of medical devices. It also supports decision-making at the national and regional levels and produces HTA reports for the Ministry of Health. In addition to the AGENAS, there is a broad network of regional and hospital-based HTAs in Italy with the objective of informing the controlled introduction of medical technologies at the regional level.

In the Netherlands, the positions of the Dutch Healthcare Institute (Zorginstituut Nederland, ZIN) on coverage of medical technologies under the basic health insurance were screened. The ZIN position is based on assessing clinical and economic evidence of technology to determine conformity of care with the “state of science and practice”. Medical care, including expensive medications, is reviewed in accordance with criteria of necessity, effectiveness (conformity with “state of science and practice”), cost-effectiveness, and feasibility (only in case of review before the implementation of the method).

In Sweden, the search for relevant HTAs was performed in several agencies at the national (Swedish Agency for Health Technology Assessment and Assessments of Social Services and Dental and Pharmaceutical Benefits Agency) and regional/hospital level (South East Healthcare Region, HTA Skåne of the Skåne University Hospital, HTA Center of the Sahlgrenska University Hospital, HTA Center of the Stockholm County Council, Centre for Assessment of Medical Technology in Örebro, Scientific Council of the Region Dalarna). The Swedish Agency for Health Technology Assessment and Assessments of Social Services (SBU) conducts independent assessments of interventions and methods used in healthcare, dental care, and social services. The Dental and Pharmaceutical Benefits Agency (TLV) conducts health economic evaluations, which analyze the benefits and costs of medical devices in comparison to relevant alternatives, based on Swedish conditions. Several Swedish healthcare regions have established regional systems for managing the introduction of medical technologies by reviewing new methods before their implementation in healthcare practice.

In Switzerland, the publications of the Swiss Federal Office of Public Health (FOPH) and Swiss Medical Board (SMB) were screened.

In the United Kingdom, the search was conducted in relevant national agencies (National Institute for Health and Care Excellence, National Institute for Health and Care Research), as well as HTA agencies of Scotland (Scottish Health Technologies Group) and Wales (Health Technology Wales).

- The leading organization of health technology assessment is the National Institute for Health and Care Excellence (NICE). It has a crucial role in the acceptance (mainly) and reimbursement (only the Technology Appraisals program) of medical technologies. Assessments are based on a thorough review of clinical and (in some programs) economic evidence for medical technologies. The following Med Tech-related HTA programs by NICE were considered for the analysis: Medical technologies guidance, Health technology evaluation (Early Value Assessment and Late-Stage Assessment), Medtech innovation briefing (discontinued in April 2023), Diagnostics guidance, Technology Appraisals, Interventional Procedure Guidance. Clinical guidelines were not considered under the scope of the analysis.
- The National Institute for Health and Care Research (NIHR) funds valuable independent research for health and social care decision-makers. The NIHR Health Technology Assessment (HTA) Programme supports research that is immediately useful to patients, clinical practice, and policy or decision-makers. Reports from the HTA Programme are published in the NIHR HTA Journal and inform the National Institute for Health and Care Excellence (NICE) guidance.
- The Scottish Health Technologies Group (SHTG) is a national health technology assessment (HTA) agency providing advice to NHS Scotland on the use of new and existing health technologies (excluding medicines), likely to have a significant impact on healthcare. The screening of the following SHTG HTA products was conducted: SHTG Recommendation (evidence-based recommendations which consider clinical effectiveness, safety, and cost-effectiveness evidence on technology), SHTG Assessment (targeted analysis to provide support for decision-making across health and social care in Scotland), and SHTG Innovative Medical Technology Overview (light-touch overview of emerging evidence for innovative technologies).
- Health Technology Wales (HTW) is a national body working to improve the quality of care in Wales. It is funded by the Welsh Government and hosted within NHS Wales but is independent of both. HTW uses a staged process of assessment of technologies by developing three types of documents: topic exploration report (TER), Evidence Appraisal Report (EAR) and Guidance (GUI). The topic exploration report (TER) aimed to assess whether there is enough evidence to proceed with full appraisal and whether the topic meets the appraisal selection criteria. Based on the TER conclusions, HTW's Assessment Group decides whether to progress this topic further. If yes, the following two documents can be developed: Evidence Appraisal Report (EAR) and Guidance (GUI). Recommendations are not mandatory; the status of HTW guidance is "adopt or justify," meaning that the local health board and other relevant bodies are expected to report on how they have considered the appraisal and guidance. If they have chosen not to adopt HTW guidance, they are asked to outline their rationale and justify their decision.

**Supplementary Results: Results for review of health technology assessments (HTAs) of ambulatory cardiac monitoring (ACM) in Europe**

# **France**

The French National Authority for Health (HAS) published no HTAs for ambulatory cardiac monitoring during the studied period.

A total of 17 opinions of the National Committee for the Evaluation of Medical Devices and Health Technologies (CNEDiMTS) on individual implantable cardiac monitors were identified: 12 requests for the device's inclusion in the LPPR, three requests for the renewal of LPPR registration, and two requests for modification of registration conditions.

| Country | Agency | Title of report | Year | Conclusion | Reference |
| --- | --- | --- | --- | --- | --- |
| France | French National Authority for Health (HAS) | CNEDiMTS opinion for the request for the modification of registration conditions of the implantable cardiac monitor REVEAL XT by Medtronic in the LPPR | 2015 | Sufficient Actual Benefit (SA) in the etiological diagnosis of ischemic stroke and level III (moderate improvement) of Clinical Added Value (ASA) compared to the diagnostic strategy without an implantable cardiac monitor or remote monitoring. | [Link](https://www.has-sante.fr/jcms/c_2039664/fr/reveal-xt) |
| France | French National Authority for Health (HAS) | CNEDiMTS opinion for the request for the registration of the implantable cardiac monitor CONFIRM (model DM2102) by St. Jude Medical in the LPPR | 2017 | Sufficient Actual Benefit (SA) in the etiological diagnosis of recurrent unexplained syncope and ischemic stroke and level V (no improvement) of Clinical Added Value (ASA) compared to implantable cardiac monitor REVEAL XT. | [Link](https://www.has-sante.fr/jcms/c_2761859/fr/confirm-modele-dm2102) |
| France | French National Authority for Health (HAS) | CNEDiMTS opinion for the request for the registration of the implantable cardiac monitor CONFIRM (model DM3500) by Abbott in the LPPR | 2019 | Sufficient Actual Benefit (SA) in the etiological diagnosis of recurrent unexplained syncope and ischemic stroke and level V (no improvement) compared to the implantable cardiac monitor CONFIRM model DM2102. | [Link](https://www.has-sante.fr/jcms/p_3103871/fr/confirm-rx-modele-dm3500) |
| France | French National Authority for Health (HAS) | CNEDiMTS opinion for the request for the registration of the implantable cardiac monitor with remote monitoring BIOMONITOR 2-AF by Biotronik in the LPPR | 2019 | Sufficient Actual Benefit (SA) in the etiological diagnosis of recurrent unexplained syncope and ischemic stroke, and level V (no improvement) compared to other implantable cardiac monitors without remote monitoring, already supported in the selected indications. | [Link](https://www.has-sante.fr/jcms/p_3101054/fr/biomonitor-2-af) |
| France | French National Authority for Health (HAS) | CNEDiMTS opinion for the request for the modification of registration conditions of the implantable cardiac monitor CONFIRM (model DM3500) by Abbott in the LPPR | 2020 | Sufficient Actual Benefit (SA) in the etiological diagnosis of recurrent unexplained syncope and ischemic stroke, and level V (no improvement) compared to implantable cardiac monitors REVEAL LINQ and BIOMONITOR III. | [Link](https://www.has-sante.fr/jcms/p_3215295/fr/confirm-rx-modele-dm3500) |
| France | French National Authority for Health (HAS) | CNEDiMTS opinion for the request for the registration of the implantable cardiac monitor with remote monitoring BIOMONITOR III by Biotronik in the LPPR | 2020 | Sufficient Actual Benefit (SA) in the etiological diagnosis of recurrent unexplained syncope and ischemic stroke and level V (no improvement) compared to implantable cardiac monitor REVEAL LINQ. | [Link](https://www.has-sante.fr/jcms/p_3165697/fr/biomonitor-iii) |
| France | French National Authority for Health (HAS) | CNEDiMTS opinion for the request for renewal of registration of the implantable cardiac monitor REVEAL XT by Medtronic in the LPPR | 2021 | Insufficient Actual Benefit (SA): despite the available clinical data, given the clinical implications of the technical differences between REVEAL XT and REVEAL LINQ, the benefit of REVEAL XT is no longer established for the etiological diagnosis of recurrent unexplained syncope and cryptogenic ischemic stroke after initial clinical evaluation. | [Link](https://www.has-sante.fr/jcms/p_3280880/fr/reveal-xt) |
| France | French National Authority for Health (HAS) | CNEDiMTS opinion for the request for the registration of the implantable cardiac monitor with remote monitoring BIOMONITOR IIIM by Biotronik in the LPPR | 2021 | Sufficient Actual Benefit (SA) in the etiological diagnosis of recurrent unexplained syncope and ischemic stroke, and level V (no improvement) compared to the implantable cardiac monitor BIOMONITOR III. | [Link](https://www.has-sante.fr/jcms/p_3239040/fr/biomonitor-iiim) |
| France | French National Authority for Health (HAS) | CNEDiMTS opinion for the request for renewal of registration of the implantable cardiac monitor CONFIRM (model DM3500) by Abbott in the LPPR | 2023 | Sufficient Actual Benefit (SA) in the etiological diagnosis of recurrent unexplained syncope and ischemic stroke and level V (no improvement) compared to other implantable cardiac monitors registered in the LPPR. | [Link](https://www.has-sante.fr/jcms/p_3418325/fr/confirm-rx-modele-dm3500) |
| France | French National Authority for Health (HAS) | CNEDiMTS opinion for the request for the registration of the implantable cardiac monitor LINQ II by Medtronic in the LPPR | 2023 | Sufficient Actual Benefit (SA) in the etiological diagnosis of recurrent unexplained syncope and ischemic stroke, and level IV (minor improvement) compared to other implantable cardiac monitors registered in the LPPR. | [Link](https://www.has-sante.fr/jcms/p_3488412/fr/linq-ii) |
| France | French National Authority for Health (HAS) | CNEDiMTS opinion for the request for the registration of the implantable cardiac monitor JOT DX by Abbott in the LPPR | 2023 | Sufficient Actual Benefit (SA) in the etiological diagnosis of recurrent unexplained syncope and ischemic stroke and level V (no improvement) compared to the implantable cardiac monitor CONFIRM RX model DM3500. | [Link](https://www.has-sante.fr/jcms/p_3421885/fr/jot-dx) |
| France | French National Authority for Health (HAS) | CNEDiMTS opinion for the request for the registration of the implantable cardiac monitor ASSERT-IQ EL+ by Abbott in the LPPR | 2024 | Insufficient Actual Benefit (SA): the low level of evidence of the data provided does not allow the effectiveness of ASSERT-IQ EL+ to be determined. The information submitted did not enable the CNEDiMTS to assess whether data from earlier-generation implantable cardiac monitors could be extrapolated to ASSERT-IQ EL+. Given these factors, the CNEDiMTS considers that the therapeutic benefit of ASSERT-IQ EL+ cannot be established. | [Link](https://www.has-sante.fr/jcms/p_3585488/fr/assert-iq-el) |
| France | French National Authority for Health (HAS) | CNEDiMTS opinion for the request for the registration of the implantable cardiac monitor ASSERT-IQ 3+ by Abbott in the LPPR | 2024 | Insufficient Actual Benefit (SA): the low level of evidence of the data provided does not allow the effectiveness of ASSERT-IQ 3+ to be determined. The information submitted did not enable the CNEDiMTS to assess whether data from earlier-generation implantable cardiac monitors could be extrapolated to ASSERT-IQ 3+. Given these factors, the CNEDiMTS considers that the therapeutic benefit of ASSERT-IQ 3+ cannot be established. | [Link](https://www.has-sante.fr/jcms/p_3585498/fr/assert-iq-3) |
| France | French National Authority for Health (HAS) | CNEDiMTS opinion for the request for the registration of the implantable cardiac monitor BIOMONITOR IV by Biotronik in the LPPR | 2024 | Sufficient Actual Benefit (SA) in the etiological diagnosis of recurrent unexplained syncope and ischemic stroke and level V (no improvement) of Clinical Added Value (ASA) compared to implantable cardiac monitor BIOMONITOR IV. | [Link](https://www.has-sante.fr/jcms/p_3528793/fr/biomonitor-iv) |
| France | French National Authority for Health (HAS) | CNEDiMTS opinion for the request for the registration of the implantable cardiac monitor LUX-DX by Boston Scientific in the LPPR | 2024 | Insufficient Actual Benefit (SA): the low level of evidence of the data provided does not allow the effectiveness of LUX-DX to be determined. The information submitted did not enable the CNEDiMTS to assess whether the data from other ICMs could be extrapolated to LUX-DX. In view of these factors, the CNEDiMTS considers that the therapeutic benefit of LUX-DX cannot be established. | [Link](https://www.has-sante.fr/jcms/p_3531478/fr/lux-dx) |
| France | French National Authority for Health (HAS) | CNEDiMTS opinion for the request for the registration of the implantable cardiac monitor LUX-DX by Boston Scientific in the LPPR | 2025 | Sufficient Actual Benefit (SA) in the etiological diagnosis of recurrent unexplained syncope and ischemic stroke and level V (no improvement) of Clinical Added Value (ASA) compared to other implantable cardiac monitors registered in the LPPR. | [Link](https://www.has-sante.fr/jcms/p_3588820/fr/lux-dx) |
| France | French National Authority for Health (HAS) | CNEDiMTS opinion for the request for renewal of registration of the implantable cardiac monitor REVEAL LINQ by Medtronic in the LPPR | 2025 | Sufficient Actual Benefit (SA) in the etiological diagnosis of recurrent unexplained syncope and ischemic stroke and level V (no improvement) of Clinical Added Value (ASA) compared to other implantable cardiac monitors registered in the LPPR. | [Link](https://www.has-sante.fr/jcms/p_3585490/fr/reveal-linq) |

**Germany**

The Institute for Quality and Efficiency in Healthcare (IQWiG) published no HTAs for ambulatory cardiac monitoring during the studied period.

**Italy**

One Horizon Scanning report by the National Agency for Regional Health Services (AGENAS) on a portable single-channel ECG recorder for smartphone was identified during the studied period.

No assessments for the ambulatory cardiac monitoring by the Lombardy HTA Program for medical devices and the Tuscany Regional Working Group on Medical Devices were identified during the studied period.

| Country | Agency | Title of report | Year | Conclusion | Reference |
| --- | --- | --- | --- | --- | --- |
| Italy | AGENAS | Portable single-channel ECG recorder for smartphone | 2015 | The available evidence shows that such a device seems reliable in recording the ECG tracings when compared with the traditional 12-lead ECG or traditional transtelephonic monitor (TTM) with a high level of sensitivity and specificity (diagnostic test accuracy) for AF. Besides, it seems also trustworthy in measuring other cardiac parameters/conditions (QT interval, QRS delay, AV block). With limited data from ongoing trials, especially for applications in the Italian National Health System, further evidence is needed before widespread clinical adoption, particularly due to potential impacts on healthcare organizations and costs. | [Link](https://www.agenas.gov.it/images/agenas/hta/Report_HS_definitivo/19/Report_HS19_ingl.pdf) |

**Netherlands**

No assessments of ambulatory cardiac monitoring by the Dutch Healthcare Institute (ZIN) were identified during the studied period.

**Sweden**

In total, three published Swedish Agency for Health Technology Assessment and Assessments of Social Services (SBU) assessments of ambulatory cardiac monitoring devices were identified. These reports consider thumb ECG and long-term heart rhythm recording for arrhythmia detection and are described as a scientific basis for the recommendations or guidelines of the Swedish National Board of Health and Welfare. No ongoing assessments of ambulatory cardiac monitoring devices by the SBU were identified.

Six assessments of ambulatory cardiac monitoring devices by the Dental and Pharmaceutical Benefits Agency (TLV) were identified; of these, four assessments were published and two were discontinued. The technologies considered in the published evaluations included thumb ECG, KardiaMobile, KardiaApp, KardiaPro, Coala Heart Monitor Pro, and Zenicor-ECG. The technologies for which the development of assessments was discontinued included PhysioMem PM 100, CardioMem CM 100 XT, and PhysioGate App. No ongoing assessments of ambulatory cardiac monitoring devices by the TLV were identified.

One assessment of the self-monitoring of heart rhythm with Coala Heart Monitor®, developed by the Regional Methods Council of the South East Healthcare Region, was identified.

No published or ongoing assessments of ambulatory cardiac monitoring devices by other Swedish regions (HTA Skåne of the Skåne University Hospital, HTA Center of the Sahlgrenska University Hospital, HTA Center of the Stockholm County Council, Centre for Assessment of Medical Technology in Örebro, Scientific Council of the Region Dalarna) were identified.

| Country | Agency | Title of report | Year | Conclusion | Reference |
| --- | --- | --- | --- | --- | --- |
| Sweden | Swedish Agency for Health Technology Assessment and Assessments of Social Services (SBU) | Screening for atrial fibrillation with a thumb ECG for the prevention of stroke (scientific basis) | 2017 | In Sweden, a thumb ECG performed twice daily for 2-4 weeks and during episodes of perceived irregular heartbeat detects previously unknown AF in approximately 3% of individuals aged 75.  For AF screening in individuals aged 75 years, corresponding to a stroke risk level (CHA₂DS₂-VASc) of at least 2, thumb ECG has been evaluated in small studies (≤108 participants). One study involving 100 patients reported a sensitivity of 94% and a specificity of 92% for detecting AF using thumb ECG. Based on an unadjusted benefit–risk analysis, it is estimated that preventive treatment could reduce the annual number of cerebral infarctions versus cerebral hemorrhages by approximately 60 to 92 cases, assuming equal treatment effectiveness in screen-detected and clinically diagnosed AF.  In patients aged 70-80 with known AF (detected due to symptoms or other reasons) and a CHA₂DS₂-VASc score of 3 or 4, the estimated annual net benefit (benefit–risk balance) is +2.4% to +4.0%, unadjusted for the severity difference between cerebral infarction and hemorrhage, and +1.1% to +1.4% when such severity differences are considered. | [Link](https://www.sbu.se/sv/publikationer/sbu-bereder/screening-for-formaksflimmer-med-tum-ekg-i-syfte-att-forebygga-stroke/) |
| Sweden | Swedish Agency for Health Technology Assessment and Assessments of Social Services (SBU) | Scientific basis for the National Board of Health and Welfare’s national guidelines for stroke care | 2016 | Long-term heart rhythm recording for arrhythmia detection, in addition to routine ECG, has an unclear effect on the diagnosis of AF.  The identified review included only studies with low evidence value due to poor design and a high risk of bias. No other acceptable studies published after the review’s date could be identified. The SBU assessed that this evidence is insufficient to form a scientific basis. | [Link](https://www.sbu.se/sv/publikationer/sbu-bereder/vetenskapligt-underlag-till-socialstyrelsens-nationella-riktlinjer-for-strokesjukvard/) |
| Sweden | Swedish Agency for Health Technology Assessment and Assessments of Social Services (SBU) | Update of documentation for the National Board of Health and Welfare’s national guidelines for stroke care | 2018 | In patients with cryptogenic ischemic stroke or transient ischemic attack, screening for arrhythmia via long-term recording of the heart rhythm in addition to routine ECG, compared with screening with routine ECG alone, results in a moderate increase in detection of paroxysmal AF.  This conclusion is based on one acceptable-quality systematic review and one complementary study published after the review's search date, with a medium risk of bias. The total number of participants was 1,547, distributed across five studies. The review did not include a detailed quality review of the included studies. | [Link](https://www.sbu.se/sv/publikationer/sbu-bereder/uppdatering-av-underlag-till-socialstyrelsens-nationella-riktlinjer-for-strokesjukvard/) |
| Sweden | Dental and Pharmaceutical Benefits Agency (TLV) | Knowledge base: Health economic evaluation of primary preventive screening of atrial fibrillation with thumb ECG | 2016 | The optimal age for AF screening, from a cost-effectiveness standpoint, is 75 years for both men and women. The cost per quality-adjusted life year (QALY) gained is estimated at SEK 7,000 for women and SEK 91,000 for men. However, the cost per QALY increases with the number of screenings performed.  A key uncertainty lies in the proportion of silent (asymptomatic) AF that can be detected through screening. There are currently no studies comparing screening outcomes across different age groups, between genders, or evaluating the effect of repeated (e.g., twice) screening rounds. | [Link](https://www.tlv.se/medicinteknikforetag/halsoekonomiska-bedomningar-och-rapporter-medicintekniska-produkter/avslutade-bedomningar/2016-05-04-utvidgat-kunskapsunderlag-av-tum-ekg.html) |
| Sweden | Dental and Pharmaceutical Benefits Agency (TLV) | Health economic evaluation of KardiaMobile, KardiaApp, and KardiaPro in atrial fibrillation | 2022 | The cost-effectiveness of KardiaMobile compared to a 24-hour Holter ECG was evaluated through both cost-impact and cost-effectiveness analyses. The TLV estimated that the proportion of primary stroke events potentially avoided ranged from 0% to 0.67%, with associated cost savings between 293 SEK and 10,128 SEK compared to 24-hour Holter monitoring.  KardiaMobile may offer greater clinical benefit at a lower cost. Even in scenarios where no stroke events are prevented, KardiaMobile remains cost-saving relative to a 24-hour Holter ECG, assuming the examination costs, including healthcare resource utilization, align with the analysis assumptions. | [Link](https://www.tlv.se/medicinteknikforetag/halsoekonomiska-bedomningar-och-rapporter-medicintekniska-produkter/avslutade-bedomningar/2022-02-07-halsoekonomisk-bedomning-av-kardiamobile-vid-formaksflimmer.html) |
| Sweden | Dental and Pharmaceutical Benefits Agency (TLV) | Health economic evaluation of Coala Heart Monitor Pro in atrial fibrillation | 2022 | The cost-effectiveness of the Coala Heart Monitor Pro compared to a 24-hour Holter ECG was evaluated through a cost-benefit analysis, focusing on its ability to detect AF over a 14-day monitoring period. TLV estimated a QALY gain between 0 and 0.0024 and cost savings of 606-929 SEK compared with a 24-hour Holter ECG.  The Coala Heart Monitor Pro may offer greater benefits at a lower cost. Even in scenarios where no QALY gain is achieved, the analysis indicates that use of the Coala Heart Monitor Pro remains cost-saving compared to Holter ECG, provided that the costs align with those used in the analysis. | [Link](https://www.tlv.se/medicinteknikforetag/halsoekonomiska-bedomningar-och-rapporter-medicintekniska-produkter/avslutade-bedomningar/2022-02-07-halsoekonomisk-bedomning-av-coala-heart-monitor-pro-vid-formaksflimmer.html) |
| Sweden | Dental and Pharmaceutical Benefits Agency (TLV) | Health economic evaluation of Zenicor-ECG in atrial fibrillation | 2022 | The cost-effectiveness of Zenicor-ECG compared to 24-hour Holter ECG was evaluated through a cost-benefit analysis, focusing on its ability to detect AF over a 14-day monitoring period. In the base-case scenario, TLV estimated an average QALY gain of 0.0037 and a cost saving of approximately SEK 384 compared to a 24-hour Holter ECG.  The base-case health economic analysis suggests that Zenicor-ECG offers greater clinical benefit at a lower cost  The level of uncertainty in the economic evaluation is considered medium to high; however, this uncertainty has been addressed through sensitivity analyses and does not alter the conclusion regarding the device's cost-effectiveness. | [Link](https://www.tlv.se/medicinteknikforetag/halsoekonomiska-bedomningar-och-rapporter-medicintekniska-produkter/avslutade-bedomningar/2022-02-07-halsoekonomisk-bedomning-av-zenicor-ekg-vid-formaksflimmer.html) |
| Sweden | Dental and Pharmaceutical Benefits Agency (TLV) | Assessment of CardioMem CM 100 XT and PhysioGate App | 2022 | The health economic assessment was not performed due to the lack of published clinical studies on CardioMem CM 100 XT demonstrating clinical efficacy/patient benefit. | [Link](https://www.tlv.se/medicinteknikforetag/halsoekonomiska-bedomningar-och-rapporter-medicintekniska-produkter/avslutade-bedomningar/2022-02-07-bedomning-av-cardiomem-cm-100-xt-och-physiogate-app.html) |
| Sweden | Dental and Pharmaceutical Benefits Agency (TLV) | PhysioMem PM 100 for atrial fibrillation | 2022 | GETEMED Medizin- und Informationstechnik AG declined to provide data, so TLV did not proceed with the assessment. | [Link](https://www.tlv.se/medicinteknikforetag/halsoekonomiska-bedomningar-och-rapporter-medicintekniska-produkter/avslutade-bedomningar/2022-02-07-physiomem-pm-100-vid-formaksflimmer---foretaget-valde-att-inte-medverka-med-underlag.html) |
| Sweden | Regional Methods Council, South East Healthcare Region | Self-monitoring of heart rhythm with Coala Heart Monitor® | 2021 | Three scientific abstracts (2018-2019) reported data from a study of 1,000 randomly selected ECGs recorded with the Coala device: compared to blinded cardiologist interpretation, the combination of thumb and chest ECGs showed a higher positive predictive value (0.872) than thumb-ECG alone (0.647); however, 13% of interpretations were still false positive. Sensitivity for AF detection was 0.951; specificity was 0.976.  There were no cost-effectiveness studies on Coala. Nonetheless, given the improved diagnostic accuracy reported in abstracts, it is considered potentially cost-effective, assuming better preventive treatment of AF leads to reduced stroke incidence and improved health outcomes. Cost-effectiveness depends on AF risk in the tested population. | [Link](https://sydostrasjukvardsregionen.se/samverkansgrupper/hta/genomforda-bedomningar/) |

**Switzerland**

No HTA reports dedicated to ambulatory cardiac monitoring devices were identified within the HTA program of the Swiss Federal Office of Public Health (FOPH) and Swiss Medical Board (SMB).

Annex 1 of the Health Benefits List (Krankenpflege-Leistungsverordnung, KLV) lists explicitly evaluated services and indicates their coverage status under compulsory health insurance. Verification of services in terms of clinical effectiveness, appropriateness, and cost-effectiveness is performed by the Medical Benefit Division (part of FOPH). Two technologies associated with ambulatory cardiac monitoring are included in Annex 1 of the KLV list with the status “Yes” (continue coverage).

| Country | Agency | Title of report | Year | Conclusion | Reference |
| --- | --- | --- | --- | --- | --- |
| Switzerland | Federal Office of Public Health (Medical Benefit Division) | Long-term ECG monitoring | 2020 | The technology “Long-term ECG monitoring” was evaluated by the Federal Office for Public Health and included in Annex 1 of KLV with the status “Yes” (continue coverage) since 13.05.1976. The main indications are rhythm and conduction disturbances, as well as myocardial circulatory disorders (coronary disease). The device can also be used to monitor treatment efficacy. | [Link](https://www.bag.admin.ch/bag/de/home/versicherungen/krankenversicherung/krankenversicherung-leistungen-tarife/Aerztliche-Leistungen-in-der-Krankenversicherung/anhang1klv.html) |
| Switzerland | Federal Office of Public Health (Medical Benefit Division) | Subcutaneous implantable loop recorder system for electrocardiogram registration | 2020 | The technology “Subcutaneous implantable loop recorder system for electrocardiogram registration” was evaluated by the Federal Office for Public Health and included in Annex 1 of the KLV with the status “Yes” (continue coverage) since 01.01.2001 / 01.01.2018. | [Link](https://www.bag.admin.ch/bag/de/home/versicherungen/krankenversicherung/krankenversicherung-leistungen-tarife/Aerztliche-Leistungen-in-der-Krankenversicherung/anhang1klv.html) |

**United Kingdom**

In total, in the United Kingdom, 19 HTAs for ambulatory cardiac monitoring devices were identified.

Eight HTAs for ambulatory cardiac monitoring by NICE were identified (six published and two ongoing): three medical technologies guidances, two health technology evaluations (Early Value Assessment), two diagnostics guidances, and one Medtech innovation briefing. The HTA reports were provided for ECG devices (including AI-assisted devices, wearable ambulatory devices with single or multiple leads), and implantable cardiac monitors.

Among the NIHR HTA programme reports, three reports for wearable ECG devices and implantable cardiac monitors were identified.

Four HTAs by the Scottish Health Technologies Group (SHTG) were identified for ambulatory cardiac monitoring (three published reports and one ongoing report). The reports evaluated different types of ECG devices (Holter devices, event recorder technologies, and patch technologies).

No guidance (full appraisals) of the ambulatory cardiac devices published by the Health Technology Wales (HTW) were identified. Four topic exploration reports (TER) were provided, three of which are published without further development of the full appraisal, and one TER with the ongoing process of the full appraisal. The reports evaluated wearable ECG devices and the digital application that uses photo-plethysmography method for the detection of atrial fibrillation.

| Country | Agency | Title of report | Year | Conclusion | Reference |
| --- | --- | --- | --- | --- | --- |
| England | National Institute for Health and Care Excellence (NICE) | Artificial Intelligence assisted Echocardiography to support diagnosis of heart failure: early value assessment (Health technology evaluation) | Ongoing | The topic is awaiting development. In April 2025, the Prioritization Board published a favorable prioritization decision (of March 2025) on evaluating AI-assisted echocardiography to support the diagnosis of heart failure. The board recognized the potential system benefits of an AI-enhanced echocardiogram, including improved diagnosis accuracy and reduced waiting times. | [Link](https://www.nice.org.uk/guidance/awaiting-development/gid-hte10067) |
| England | National Institute for Health and Care Excellence (NICE) | KardiaMobile for detecting atrial fibrillation (Medical technologies guidance) | 2022 | NICE recommended KardiaMobile as an option for detecting atrial fibrillation (AF) for people with suspected paroxysmal AF, who present with symptoms such as palpitations and are referred for ambulatory ECG monitoring by a clinician. | [Link](https://www.nice.org.uk/guidance/mtg64) |
| England | National Institute for Health and Care Excellence (NICE) | Zio XT for detecting cardiac arrhythmias (Medical technologies guidance) | 2020 | Zio XT is recommended for people with suspected cardiac arrhythmias who would benefit from ECG monitoring for longer than 24 hours, but only if NHS organizations collect data on its resource use and long-term clinical outcomes, such as hospitalization rates, etc. Evidence suggests Zio XT is more convenient and has a higher diagnostic yield than standard 24-hour Holter monitoring, with potential cost savings, though further data is required. | [Link](https://www.nice.org.uk/guidance/mtg52) |
| England | National Institute for Health and Care Excellence (NICE) | Zio XT for detecting cardiac arrhythmias (Medical technologies guidance) | Ongoing | An update to the existing MTG for Zio XT for detecting cardiac arrhythmias (2020) is suspended due to a delay with the manufacturer's submission, caused by the delay in the trial data reporting. | [Link](https://www.nice.org.uk/guidance/indevelopment/gid-mt591) |
| England | National Institute for Health and Care Excellence (NICE) | KardiaMobile 6L for measuring cardiac QT interval in adults having antipsychotic medication: early value assessment (Health technology evaluation) | 2023 | NICE recommended KardiaMobile 6L for use in psychiatric services as an option to measure cardiac QT interval for people having or about to have antipsychotic medication during evidence-generation period only if a repeat QT interval measurement using a 12‑lead ECG device is offered to specific patient groups, with training for healthcare specialists and the provision of information for patients about the testing and the possibility of repeated testing with 12‑lead device after using KardiaMobile 6L. | [Link](https://www.nice.org.uk/guidance/hte10) |
| England | National Institute for Health and Care Excellence (NICE) | Carnation Ambulatory Monitor for ambulatory detection of cardiac arrhythmias (Medtech innovation briefing) | 2021 | The device's innovative aspects include a low noise floor, which leads to more accurate and more precise detection, and prolonged monitoring for up to 14 days. The evidence from four studies showed that the device is at least as effective as other ambulatory cardiac monitors in adults with suspected arrhythmias. Key uncertainties around the evidence include the small study sample sizes. | [Link](https://www.nice.org.uk/advice/mib276) |
| England | National Institute for Health and Care Excellence (NICE) | Lead-I ECG devices for detecting symptomatic atrial fibrillation using single time point testing in primary care (Diagnostics guidance) | 2019 | There is not enough evidence for lead-I ECG devices (imPulse, Kardia Mobile, MyDiagnostick, and Zenicor-ECG) to detect atrial fibrillation when used for single-time point testing in primary care for people with signs of AF and an irregular pulse. Further research is recommended on the number of detected people with AF, how it affects primary and secondary care services, and how ECGs by the devices would be interpreted in practice. | [Link](https://www.nice.org.uk/guidance/dg35) |
| England | National Institute for Health and Care Excellence (NICE) | Implantable cardiac monitors to detect atrial fibrillation after cryptogenic stroke (Diagnostics guidance) | 2020 | Reveal LINQ is recommended as an option to help detect AF after cryptogenic stroke, including transient ischemic attacks (TIA), only if non-invasive ECG monitoring has been done and a cardiac arrhythmic cause of stroke is still suspected. Further research is recommended on BioMonitor 2‑AF or Confirm Rx to assess the diagnostic yield of these devices for AF when used in people who have had a cryptogenic stroke. | [Link](https://www.nice.org.uk/guidance/dg41) |
| England | National Institute for Health and Care Research (NIHR) | KardiaMobile 6L for measuring QT interval in people having antipsychotic medication to inform early value assessment: a systematic review | 2024 | There is insufficient evidence to support a full diagnostic assessment evaluating the clinical and cost-effectiveness of KardiaMobile 6L in the context of QT interval-based cardiac risk assessment for service users who require antipsychotic medication. The evidence to inform the aims of this early value assessment was also limited. The report includes a comprehensive list of research recommendations, both to reduce the uncertainty around this early value assessment and to provide the additional data needed to inform a full diagnostic assessment, including cost-effectiveness modeling. | [Link](https://www.journalslibrary.nihr.ac.uk/hta/TFHU0078#/abstract) |
| England | National Institute for Health and Care Research (NIHR) | Lead-I ECG for detecting atrial fibrillation in patients with an irregular pulse using single time point testing: a systematic review and economic evaluation | 2020 | Given the assumptions used in the base-case model, single-time point lead-I ECG devices for the detection of AF in people with signs or symptoms of AF and an irregular pulse appear to be a cost-effective use of NHS resources compared with manual pulse palpation (MPP) followed by a 12-lead ECG in primary or secondary care. | [Link](https://www.journalslibrary.nihr.ac.uk/hta/HTA24030) |
| England | National Institute for Health and Care Research (NIHR) | Implantable cardiac monitors to detect atrial fibrillation after cryptogenic stroke: a systematic review and economic evaluation | 2020 | Three implantable cardiac monitors included in the research (BioMonitor 2-AF, Confirm Rx, and Reveal LINQ) could be considered cost-effective at a £20,000–30,000 threshold, compared with standard of care monitoring, for cryptogenic stroke patients with no atrial fibrillation detected after 24 hours of external electrocardiographic monitoring. Further clinical studies are required to confirm their efficacy in cryptogenic stroke patients. | [Link](https://www.journalslibrary.nihr.ac.uk/hta/HTA24050) |
| Scotland | Scottish Health Technology Group (SHTG) | KardiaMobile for detecting atrial fibrillation | 2022 | SHTG recommended single-lead KardiaMobile as an option for detecting AF for people with suspected paroxysmal AF who present with symptoms such as palpitations and are referred for ambulatory ECG monitoring by a clinician. The SHTG recommendation is based on guidance produced by NICE in 2022. | [Link](https://shtg.scot/our-advice/kardiamobile-for-detecting-atrial-fibrillation/) |
| Scotland | Scottish Health Technology Group (SHTG) | Electrocardiogram (ECG) patch monitors | 2024 | The identified evidence suggests ECG patch monitors offer a convenient and comfortable alternative to traditional Holter monitors, providing extended cardiac rhythm monitoring that may improve detection of atrial fibrillation and patient compliance. However, occasional issues like skin irritation and device durability have been reported. However, concerns remain regarding diagnostic accuracy, data interpretation, and cost-effectiveness, particularly in the Scottish context, warranting further clinical validation and economic evaluation. | [Link](https://shtg.scot/our-advice/ecg-patch-monitors/) |
| Scotland | Scottish Health Technology Group (SHTG) | Detection of paroxysmal atrial fibrillation in patients with newly diagnosed ischemic stroke | 2021 | There is insufficient evidence to draw robust conclusions about the comparative clinical and cost-effectiveness of different types of devices (Holter devices, event recorder technologies, and patch technologies) for ambulatory ECG to detect paroxysmal AF. | [Link](https://shtg.scot/our-advice/detection-of-paroxysmal-atrial-fibrillation-in-patients-with-newly-diagnosed-ischaemic-stroke/) |
| Scotland | Scottish Health Technology Group (SHTG) | ECG patch monitors | Ongoing (June 2025) | The SHTG has been asked to assess the clinical effectiveness, cost-effectiveness (including system efficiencies), and safety of ECG patch monitors compared with traditional Holter monitors or cardiac event recorders in detecting paroxysmal AF. The final report is expected on June 30, 2025. | [Link](https://shtg.scot/our-advice/electrocardiogram-ecg-patch-monitors/) |
| Wales | Health Technology Wales (HTW) | Ambulatory patch electrocardiography monitoring devices used to detect  cardiac arrhythmias (Topic Exploration Report) | 2021 | Based on the identified evidence (three HTAs on two different technologies), ambulatory monitoring of patients suspected of cardiac arrhythmias achieved through patch ECG monitoring devices appears to be a suitable alternative to the current practice. Some areas of uncertainty remain to be clarified, including the target population, the difference between the ECG patch technologies and the associated diagnostic accuracies, the criteria for ECG monitoring, and the necessity of additional regulatory approval for devices embedding digital health technologies. HTW’s Assessment Group decided not to develop a full appraisal on this topic. | [Link](https://healthtechnology.wales/reports-guidance/ambulatory-patch-electrocardiography-monitoring-devices/) |
| Wales | Health Technology Wales (HTW) | Alivecor KardiaMobile for the detection of atrial fibrillation (Topic Exploration Report) | 2018 | HTW reviewed evidence regarding using the AliveCor Kardia mobile device to detect AF and reduce stroke risk in at-risk populations within primary care. However, HTW found limited direct relevance as existing studies often focused on diagnosed AF management or broader screening programs rather than primary care detection. Consequently, it remains unclear whether the available evidence explicitly supports the device's use for AF detection in primary care settings. | [Link](https://healthtechnology.wales/reports-guidance/alivecor-kardiamobile-device/) |
| Wales | Health Technology Wales (HTW) | Handheld single lead electrocardiogram devices to detect atrial fibrillation in older adults and those with intermittent episodes (Topic Exploration Report and Evidence Appraisal Report) | TER – 2020, EAR - 2021 | The HTW Assessment Group concluded that there was insufficient evidence to support full appraisal development.  Evidence suggests that handheld lead-I ECG devices have high sensitivity and specificity for detecting AF, but their benefit over simpler methods appears limited, with few studies showing improved health outcomes or cost-effectiveness. While these devices are generally well-accepted and effective in identifying intermittent AF, challenges such as low screening uptake, organizational integration, and the need for clinician oversight remain, indicating that simpler opportunistic screening approaches may be equally effective and more feasible for population-wide implementation. | [Link](https://healthtechnology.wales/reports-guidance/handheld-single-lead-electrocardiogram-devices/) |
| Wales | Health Technology Wales (HTW) | Photo-plethysmography for the management of atrial fibrillation (Topic Exploration Report) | Ongoing  (TER was published in 2024) | The report is focused on FibriCheck, a digital application that uses photoplethysmography from a smartphone camera and an AI algorithm to distinguish AF from sinus rhythm.  The identified evidence showed the device's benefits in comparison with traditional ECG methods, such as shorter hospital stays, fewer readmissions, reduced ECG usage, and lower healthcare and travel costs. However, further research is needed on the target population and care pathway for FibriCheck, as current evidence primarily comes from cohort studies focusing on people with known AF. Based on the TER conclusions, HTW's Assessment Group decided to proceed with a full appraisal on this topic. | [Link](https://healthtechnology.wales/reports-guidance/photo-plethysmography-for-the-management-of-atrial-fibrillation/) |

Supplementary Table: Reimbursement of ambulatory ECG monitoring and ILR

With the exception of the UK, the cost of the ILR device is not included in the physician reimbursement for outpatient ILR implantation, varies by country, and there is limited publicly available data. Reference pricing for ILR devices across countries ranges from €1,409 to €4296 (Globaldata.com, Report Code: GDME12777GBP-MP-L5).

| **Country** | **Ambulatory ECG monitoring** | **ILR** |
| --- | --- | --- |
| France | Outpatients  One specific code (DEQP005) for Holter monitoring via fee for service, €77.01 ^20, 46^  Day case  In hospital settings with a one-day stay or day case, Holter monitoring is allocated to the DRGs for very short stays  The DRG allocation is diagnosis-driven and differs for syncope and different types of arrhythmias   - Syncope and collapse, 05M05T, €416.14 plus CCAM fee - Arrhythmias and cardiac conduction disorders, 05M08T, €536.86 plus CCAM fee   Patch ECG monitoring is not specifically coded and reimbursed | Implantation and removal coded within the same DRG codes, although implantation and removal each have a different CCAM fee  Reading the results of the ILR is coded via a separate specific code ^20, 46-48^  DRG allocation depends on clinical diagnosis, severity levels, length of stay and in some diagnoses also depends on hospital type (ordinary, public, private, private not for profit)  2-day length of stay   - Syncope and collapse, 05M051, €1,491.55 (public hospital) and €740.47 (private hospital) - Arrhythmias and cardiac conduction disorders, 05M081, €961.63 (public hospital) and €536.86 (private hospital)   Day case or 1 day length of stay   - Syncope and collapse, 05M05T, €708.35 (public hospital) and €416.14 (private hospital) - Arrhythmias and cardiac conduction disorders, 05M08T, €961.63 (public hospital) and €536.86 (private hospital)   Severity of disease   - Syncope and collapse, 05M05, levels 2-4 attract a higher tariff up to €4,945.76 (public hospital) and €2,696.74 (private hospital) - Arrhythmias and cardiac conduction disorders, 05M08, levels 2-4 attract a higher tariff up to €4,608.27 (public hospital) and €2,506.26 (private hospital)   DRG are plus add on fees and private hospitals have an additional CCAM fees  In private for-profit hospitals, physicians receive fees associated with the CCAM code for performing specific interventions   - ECG, with triggered event recording and teletransmission, DEQP001, €14.26 - ECG with subcutaneous implantation of a continuous recording device, DEQA001, €56.53 for implantation and €28.27 for removal   Brand specific reimbursement are also available as the add on to the base DRG for six implantable monitors (five same price of €1,408.77, one different €1,188.38) |
| Germany | Reimbursed by fee for service, via EBM ^54^ codes for each element of the service   - ECG recording, 13252, €5.73 - ECG evaluation, 13253, €10.26 - Shipping of materials, 40106, €1.50   Patch ECG monitoring is not specifically coded and reimbursed in the EBM | Inpatient setting  Different OPS codes ^19^ for implantation (5-377.8) and removal (5-378.07) of ILR  Two different DRG codes for implantation and removal of ILR  The DRG tariffs^32^ depend on length of stay and complexity of diagnosis   - Implantation, F12F, €5298 - Removal, F18D, €2,879   Complex cases   - Implantation of ILR, F12A, F12B, F12C, F12D, F12E, price ranging from €8,402 to €18,718 - Removal of ILR, F18A, F18B, F18C, price ranging from €5,970 to €15,278   Additional innovation funding (NUB)^69^ is only available for ILR with simultaneous cardiac ablation grouped to the specific DRG (F50A) Day case setting  Implantation not within the EBM^54^, but removal is coded (two separate codes)  Monitoring during the operation, anesthesia, post-operative monitoring and care reimbursed separately   - Removal, 31211, €63.491or 36211, €79.36 - Monitoring during operation, 31503, €58.24 or 36503, €6.92 - Post-operative monitoring, 31601, €17.42 - Post-operative care, 31602, €10.14 - Anaesthesia, 31821, €118.98 or 36821, €68.62 |
| Italy  National costs shown, but considerable regional variation | Two codes for Holter monitoring ^40, 49-51^   - Dynamic ECG, 89.50, €61.95 or €89.54 (potentially can be used for patch ECG) - ECG monitoring, 89.54, €46.45 or €46.48   Cost variable by region | Variable coding for both implantation ^40.43^ (37.79, €1,550.45) and removal (86.05, €40.05) using nonspecific codes  OR  In inpatient and day-case setting, implantation and removal reimbursed via a DRG with cost dependent on length of stay  If patient has complications, then removal is allocated to a different DRG with a higher cost  DRGs for implantation   - Ordinary hospital stay (0-1 day)/day case, 117, €1,562 - Ordinary hospital stay (>1 day), 117, €3,547   DRGs for removal   - Ordinary hospital stay (0-1 day)/day case, 145, €220 - Ordinary hospital stay (>1 day), 145, €2,097 - Complex cases, ordinary hospital stay (0-1 day)/day case, 144, €259 - Complex cases, ordinary hospital stay (>1 day), 144, €3,910   Method of coding/reimbursement and price variable by region. |
| Netherlands | Two specific codes for Holter monitoring used in combination (039755 and 039757)^34, 35, 55-57^  DRG allocation (and cost) depends on medical specialty (cardiology/internal medicine), diagnosis and whether one or both DRG codes used   - Cardiology: diagnostics/intervention and/or more than 2 outpatient clinic visits/remote consultations in cardiology for an arrhythmia of the heart, 099899063, €515 ^35^ - Cardiology: diagnosis/intervention and/or more than 2 outpatient visits/ remote consultations in case of an arrhythmia of the heart (chamber), 099899069, €545 ^35^ - Cardiology: diagnostics/ intervention and/or more than 2 outpatient visits/ remote consultations in case of an impulse and/or conduction disorder of the heart, 099899075, €460 ^35^ - Internal medicine: treatment or examination and/or more than 2 outpatient clinic visits/remote consultations and/or a maximum of 2 days of treatment for an arrhythmia of the heart, 099899015, €735 ^35^   DRGs belong to the B-segment (free segment) of the DRG system meaning that they are determined by negotiations between insurance companies and hospitals.  When requested by Primary Care (General Practitioner), Holter monitoring is reimbursed via a fee for service model as supplementary services. Cost depends on the locality of the provider.   - Analysis of a 24-hour electrocardiography registration, 039755, €190.27-€199.44 (sample of three University Medical Centers) ^55-57^ - Assessment of ECG, Holter, exercise test, etc, 039757, €68.44-€92.32 (sample of three University Medical Centers) ^55-57^ | Two different codes for implantation (039698) and removal (039699) of ILR, device itself is coded separately and used for registration purposes (190329), rather than for reimbursement ^34, 35, 55-57^  DRG allocation (and cost) depends on setting (inpatient/day case), medical specialty (cardiology/ cardiothoracic surgery)  DRGs for implantation   - Cardiology/hospital setting, 099899054, €6555 ^35^ - Cardiology/day case setting, 099899053, €3,580 ^35^ - Cardiothoracic surgery/hospital setting, 099899121, €7,863 (sample of three University Medical Centers) - Cardiothoracic surgery day case setting, 099899122, €4,403 (sample of three University Medical Centers) ^55-57^   DRGs for removal   - Cardiology/hospital setting, 099899052, €2,484 ^35^ - Cardiology/day case setting, 099899051, €1,150 ^35^ - Cardiothoracic surgery/hospital setting, 099899123, €2,403^30^ - Cardiothoracic surgery day case setting, 099899124, €1,871 (sample of three University Medical Centers) ^55-57^   Procedure codes for ILR implantation and removal have a DRG coverage period of 42 days  DRG are B class meaning that they are determined by negotiations between insurance companies and hospitals, therefore there is regional variation in reimbursement |
| Sweden | Specific KVÅ procedure codes ^21^ for Holter monitoring (including connection, recording and assessment, when carried out at same unit)   - Long-term ECG recording, arrhythmia analysis, (Holter), AF040 - Long-term ECG recording, ST segment analysis, (Holter), AF042   Holter monitoring (assessment only), AF041  Holter monitoring (connection only), AF043  Remote assessment via telemedicine (ZV051) can be specified  Outpatient setting ^38, 52, 53^  Holter monitoring (including connection and assessment), depends on diagnosis   - Atherosclerotic heart disease, with physician visit, E520, 4,954 SEK (€436.70) - Cardiac arrhythmia, with physician visit, E650, 5,198 SEK (€458.20) - Chronic heart failure, with physician visit, E470, 5,116 SEK (€450.98)   Stand-alone assessment of long-term ECG recording   - Any diagnosis, without physician visit: Remote medical care for diseases of the circulatory system, E99Z, 2,680 SEK (€236.24) - Atherosclerotic heart disease, with physician visit, E520, 4,954 SEK (€436.70) - Cardiac arrhythmia, with physician visit, E650, 5,198 SEK (€458.20) - Chronic heart failure, with physician visit, E470, 5,116 SEK (€450.98) | Specific KVÅ procedure codes ^21^ for ILR for each element of the process: implantation (FPK00), reading (DF031) and removal (DF032)  Remote assessment via telemedicine (ZV051) can be specified  Inpatient setting ^38, 52, 53^  Implantation with/without reading and/or removal, E26E, 66,595 SEK (€5,870.36)  Complex cases   - Implantation or replacement of a permanent pacemaker or loop recorder, very complicated, E26A, 127,831 SEK (€11,268.33) - Implantation or replacement of a permanent pacemaker or loop recorder, complicated, E26C, 81,133 SEK (€7,151.89)   Outpatient setting ^38, 52, 53^  Implantation with/without reading and/or removal, E26O, 28,669 SEK (€2,527.18)  Reading/monitoring of the ILR, depends on diagnosis   - Any diagnosis, without physician visit: Remote medical care for diseases of the circulatory system, E99Z, 2,680 SEK (€236.24) - Atherosclerotic heart disease, with physician visit, E520, 4,954 SEK (€436.70) - Cardiac arrhythmia, with physician visit, E650, 5,198 SEK (€458.20) - Chronic heart failure, with physician visit, E470, 5,116 SEK (€450.98)   Removal of the ILR   - Atherosclerotic heart disease, with physician visit, E520, 4,954 SEK (€436.70) - Cardiac arrhythmia, with physician visit, E650, 5,198 SEK (€458.20) - Chronic heart failure, with physician visit, E470, 5,116 SEK (€450.98) |
| Switzerland | Reimbursed via fee for service model (TARMED) ^58^Holter reimbursement can be achieved via a combination of codes for ECG registration, assessment based on wear time and patient activated;   - Connection and removal, code 17.0130, 62.77 CHF [€64.60] - Assessment of results, code 17.0140, (8-16 hours Holter wear time), 107.55 CHF [€110.69] - Assessment of results, code 17.0150, (16-24 hours Holter wear time), 141.73 CHF [€145.86])   Patient-activated long-term ECG monitoring is reimbursed via a different combination of codes for ECG registration   - Connection and removal, code 17.0160, 94.12 CHF[€96.87]), - Surcharge for programming, code 17.0170, 53.78 CHF [€55.35]) - Assessment of results, code 17.0160, 80.67 CHF [€83.02])   In the Statement of the Tariff Commission of the Swiss Society of Cardiology issued on August 30, 2022, the TARMED representation of long-term ECG monitoring was classified. After inquiries about the economic efficiency and appropriateness of using repeated Holter ECGs, the Tariff Commission discussed and evaluated these questions and formulated a recommendation/guideline for the defined tariff use of position 17.0150. The following coding was recommended depending on the duration of monitoring:   - Holter ECG monitoring < 48 hours: 1 x Code 17.0150 - Holter ECG monitoring > 48 hours up to 6 days: 2 x Code 17.015 - Holter ECG monitoring 6 - 10 days: 3 x Code 17.0150   Under certain circumstances, the following rules can be applied for long-term ECG studies > 10 days:  o Holter ECG monitoring 11 - 20 days: 4 x Code 17.0150  o Holter ECG monitoring 21 - 30 days: 5 x Code 17.0150  o Holter ECG monitoring > 30 days: 6 x Code 17.015No specific coding for patch ECG monitoring | Inpatient setting  Two different CHOP codes for implantation (37.8C) and removal (37.8D) of ILR  The DRG tariffs ^32, 33^ depend on length of stay and complexity of diagnosis   - Implantation of ILR, F12F, 9,010 CHF (€9,272.82) for 1 day, 15,520 CHF (€15,972.72) for 2 days - Removal of ILR, F18B, 6570 CHF (€6,761.65) for 1 day, 12,920CHF (€13,296.88) for 2 days - However, according to the Healthcare Benefit Oridance (KLV), the ILR implantation and removal procedures should be performed in day case and outpatient settings (with fee for service reimbursement via TARMED)   Outpatient setting  Reimbursed via fee for service model (TARMED) ^58^  Three TARMED codes for   - Implantation,17.1670, 476.10 CHF (€489.990) - Removal, 17.1690, 342.33 CHF (€352.32) - Assessment, 17.1680 154.61 CHF (€159.12)   The cost of consumables and device is billed separately if price >3 CHF (€3.09)/item |
| UK | NHS Payment Scheme ^31^ reports one DRG (EY51Z for Electrocardiogram Monitoring or Stress Testing) with reimbursement according to setting   - Outpatient £135 (€159.42) - Day case £135 (€159.42) - Non-elective £462 (€545.58) - Per day long stay £305 (€360.17)   Reimbursement varies according to Market Forces Factor  Patch ECG monitoring is not specifically coded, but is reimbursed within EY51Z | NHS Payment Scheme ^31^ reports three DRGs (two for implantation and one for removal) for ILR and depends on complications, co-morbidity profile and setting    Implantation with complication and comorbidity score 0-2), EY12B,   - - Outpatient £2,405 (€2,840.06)   - Day case £2,405 (€2,840.06)   - Non-elective £3,896 (€4,600.79)   - Per day long stay £305 (€360.17)   Implantation with complication and comorbidity score 3+, EY12A   - - Day case £2,584 (€3,051.45)   - Non-elective £4,969 (€5,867.89)   - Per day long stay £305 (€360.17)   Removal of ILR   - - Outpatient £684 (€807.74)   - Day case £684 (€807.74)   - Non-elective £784 (€925.83)   - Per day long stay £305 (€360.17)   Reimbursement varies according to Market Forces Factor |
